# Cytosolic Sequestration of Prep1 Influences Early Stages of T Cell Development

**DOI:** 10.1371/journal.pone.0002424

**Published:** 2008-06-18

**Authors:** Dmitry Penkov, Martina Palazzolo, Anna Mondino, Francesco Blasi

**Affiliations:** 1 Molecular Genetics Unit, San Raffaele Scientific Institute and Università Vita Salute San Raffaele, Milan, Italy; 2 Lymphocyte Activation Unit, San Raffaele Scientific Institute and Università Vita Salute San Raffaele, Milan, Italy; 3 Institute of Molecular Oncology, Milan, Italy; The Babraham Institute, United Kingdom

## Abstract

**Objective:**

Prep1 and Pbx2 are the main homeodomain DNA-binding proteins of the TALE (three amino acid loop extension) family expressed in the thymus. We previously reported reduced *Pbx2* expression and defective thymocyte maturation in *Prep1* hypomorphic mice. To further investigate the role of this homeodomain DNA-binding protein in T cell development, we generated transgenic mice expressing the N-terminal fragment of Pbx1 (Pbx1NT) under the control of the Lck proximal promoter.

**Principal Findings:**

Pbx1NT causes Prep1 cytosolic sequestration, abolishes Prep1-dependent DNA-binding activity and results in reduced *Pbx2* expression in developing thymocytes. Transgenic thymi reveal increased numbers of CD4^−^ CD8^−^ CD44^−^ (DN3 and DN4) thymocytes, due to a higher frequency of DN2 and DN4 Pbx1NT thymocytes in the S phase. Transgenic thymocytes however do not accumulate at later stages, as revealed by a normal representation of CD4/CD8 double positive and single positive thymocytes, due to a higher rate of apoptotic cell death of DN4 Pbx1NT thymocytes.

**Conclusion:**

The results obtained by genetic (*Prep1* hypomorphic) and functional (Pbx1NT transgenic) inactivation of Prep1 support nonredundant roles for this homeodomain protein during different stages of T cell development.

## Introduction

Prep1 is a transcription factor belonging to the TALE (*t*hree-*a*mino-acid *l*oop *e*xtension) family of homeodomain proteins, which in vertebrates comprises the PBC (Pbx1 to Pbx4), Prep (Prep1 and Prep2) and Meis (Meis1, Meis2 and Meis3) subfamilies [1]. Prep1 is present in a cytoplasmic and a nuclear localizations in different hematopoietic precursors [2] and plays a central role starting from the earliest stages of embryonic development since a Prep1 null mutation causes lethality around gastrulation (L.C. Fernandez & F. Blasi, unpublished data). We have recently generated *Prep1* hypomorphic mice (*Prep1^i/i^*), in which the level of Prep1 is reduced to about 2–10% of wild type (wt), and found that *Prep1^i/i^* embryos die (in 80% of the cases) at E17.5-P0 with anemia and major defects in oculogenesis and angiogenesis [3], [4]. The other 20% of the embryos develop to term and live a normal-length life. *Prep1^i/i^* mice show a decreased number of single-positive thymocytes due to a higher propensity of DP (CD4^+^ CD8^+^) cells to undergo apoptotic cell death [3].

We previously demonstrated that a truncated N-terminal fragment of a Pbx1 protein lacking the homeodomain and the nuclear localization signal (Pbx1NT) preserves the ability to bind Prep1, but sequesters it into the cytoplasm [5]. To investigate the relative contribution of cytosolic and nuclear Prep1 to T cell development we generated transgenic (TG) mice expressing the *Pbx1NT* under the control of the *lck* proximal promoter. We report that *Pbx1NT* expression causes relocalization of Prep1 in the cytosol of developing thymoctes, and abrogates Prep1-dependent DNA-binding activity. As a consequence transgenic mice revealed abnormal early T cell development, revealed by increased numbers of DN cells in the S phase of the cell cycle.

## Materials and Methods

### Generation of Pbx1NT TG mice

To generate TG mice with expression of amino-terminal (dominant negative) part of the Pbx1 protein, Pbx1NT [4], a fragment of cDNA coding for amino acids 1–232 of human Pbx1 protein, was inserted into the p1017 cassette [6] under the transcriptional control of the lck proximal promoter. A NotI fragment of the construct was microinjected into fertilized oocytes of (FVB/N) mice and two transgene founders obtained. Both TG lines were backcrossed to FVB/N mice for over seven generations. 6 to 12 weeks old TG and control littermate mice were analyzed.

### Antibodies

The following antibodies were used in this study: anti-CD4 (RM4-5) conjugated to CyChrome, phycoerythrin (PE), or fluorescein isothiocyanate (FITC); anti-CD8a (53-6.7) conjugated to CyChrome or allophycocyanin (APC); anti-CD25 (7D4) conjugated to APC; anti-CD44 (IM7) conjugated to PE; anti-T-cell receptor β-chain (anti-TCRβ) (H57-597) conjugated to FITC; anti-TCRβ (H57-597) conjugated to biotin; and anti-CD16/CD32 (Fc receptor; 24G2) (BD Biosciences, San Jose, CA).

Anti-Pbx1 and anti-Pbx2, anti-HMG1 and anti-β-actin antibodies were obtained from Santa Cruz Biotechnology (Santa Cruz, CA). Anti-Prep1 (Meis4) and anti-Meis (Meis1, Meis2, and Meis3) were from Upstate Biotechnology (Upstate House, Dundee, United Kingdom). The anti-Pbx1b antibody was a generous gift from Michael Cleary. Anti-Prep1 polyclonal antibody was prepared in our laboratory [7].

The following secondary reagents were used: streptavidin-APC (BD Biosciences, San Jose, CA), streptavidin-FITC (Caltag Laboratories, Burlingame, CA), sheep anti-mouse and donkey anti-rabbit immunoglobulin G (IgG) labeled with horseradish peroxidase (HRP) (Amersham Biosciences, Amersham Place, United Kingdom), and rabbit anti-goat IgG labeled with horseradish peroxidase (Santa Cruz Biotechnology, Santa Cruz, CA).

### Flow cytometry

Cell suspensions were obtained from thymus, spleen, lymph nodes (LN) (inguinal, cervical, axillary, and brachial) by gentle disruption, passage through a cell strainer (40-µm nylon), and washing the cells with phosphate-buffered saline (PBS). Lymphocyte subsets were analyzed for detection of both surface and intracellular proteins as described previously [3].

### Thymocyte purification and cell sorting

Double-negative thymocytes were obtained by rabbit anti-CD4 (monoclonal antibody MAb 1.72) and anti-CD8 (MAb 31 M) complement-mediated lysis or purified by magnetic beads (Miltenyi Biotec, Bergisch Gladbach, Germany) when necessary. CD4^+^ CD8^+^ double-positive (DP), CD4^−^ CD8^−^ double-negative (DN), CD4^+^ and CD8^+^ single-positive (SP) subpopulations of thymocytes were sorted by a FACS Vantage cell sorter as previously described [3]. For DN1 (CD4^−^ CD8^−^ CD44^+^ CD25^−^) and DN3 cell sorting, DN thymocytes purified by magnetic beads (Miltenyi Biotec, Bergisch Gladbach, Germany), were stained with anti-CD44 and anti-CD25 antibodies and sorted by a FACS Vantage cell sorter.

### BrdU assay

Bromodeoxyuridine (BrdU) (1 mg) was injected intraperitoneally (i.p.), and the thymi were taken 2 hours after injection. Cell cycle analysis was performed with BrdU-flow kit (BD Biosciences, San Jose, CA) according to the manufacturer's recommendations.

### Quantitation of apoptosis by annexin V

Thymocytes (total or purified DN) were incubated in RPMI 1640 medium containing 10% FBS, 2 mM glutamine, 25 mM HEPES, β-mercaptoethanol, and penicillin/streptomycin at a concentration of 1×10^6^ cells (for total) or 0.2×10^6^ cells (for DN) per 200 µl in 96-well plates. Cells were stained ex-vivo or after 24 hours of incubation as described above with antibodies to CD4^+^ and CD8^+^ (in the case of total thymocytes) or with anti-CD25 and anti-CD44 antibodies (in the case of DN thymocytes). The percentage of annexin V-positive, 7-aminoactinomycin D (7AAD)-negative cells was determined with a FITC-annexin V kit (BD Biosciences, San Jose, CA) according to the manufacturer's recommendations.

### Protein extract preparation and electrophoretic mobility shift assays

Nuclear and cytoplasmic extracts from lymphocytes were purified according to a published protocol [8]. The electrophoretic mobility shift assay was performed as described previously [7]. The O1 oligonucleotide probe used (5′-CACCTGAGAGTGACAGAAGGAAGGCAGGGAG-3′) contains a binding site for Prep1-Pbx from the urokinase enhancer (underlined) [7].

### Immunoblotting

Nuclear and cytoplasmic protein extracts (30 µg each) were resolved by 8 to 10% sodium dodecyl sulfate-polyacrylamide gel electrophoresis and transferred to polyvinylidene difluoride (PVDF) membranes (Millipore, Billerica, MA) in a semi-dry blotting apparatus. The membranes were incubated with anti-Prep1 (1∶1,000), anti-Pbx1b (1∶1,000), anti-Pbx2 (1∶2,000), and anti-Meis (1∶2,000) antibodies. Blots were developed with SuperSignal Westpico or SuperSignal Westdura chemiluminescence substrate (Pierce, Rockford, IL).

### Total RNA isolation and RT-PCR

RNA was isolated from thymocytes by the TRIZOL method (Invitrogen, Carlsbad, CA) according to the manufacturer's recommendations. RNA was reverse transcribed with SuperScript II (Invitrogen) using oligo(dT) following the instructions of the manufacturer. cDNA was amplified on a GeneAmp 2400 machine (PE Applied Biosystems, Foster City, CA) under the following conditions: 1 cycle of 95°C for 3 min; indicated number of cycles at 94°C for 30 sec, 60°C for 40 sec, and 72°C for 30 sec; and 1 cycle of 72°C for 5 min. The following primer sets were used: for Prep1, 5-ACAGACGCTAAGTATAGACAG-3′ and 5′-AATCTGCTGGGATTGCACA-3′; for Pbx1, 5′-AGCTGGAGAAGTATGAGCAGGCATGC-3′ and 5′-ACTGTACATCTGACTGGCTGC-3′; for Pbx2, 5′-AGCTGGAGAAGTATGAGCAGGCATGC-3′ and 5′-GTTGGAGGTGTCAGAGTGAAC-3′; for Pbx3, 5′-GAGCTGGCCAAGAAATGCAG-3′ and 5′-GAAGATGGAGTTGTTGCGTCC-3′; for ß-actin, 5′-GGCATCCTGACCCTGAAGT-3 and 5′-CGGATGTCAACGTCACACTT-3′; for Pbx1NT, 5′-CAGAGTTTGGATGAGGCGCAGG and 5′-GCTAGGTGAGCTGTCCACAGGACCC-3′. Controls without reverse transcriptase (NO-RT controls) for each reverse transcriptase PCR (RT-PCR) were also performed.

## Results

### Generation of Lck-driven Pbx1NT TG mice

**Figure 1:**
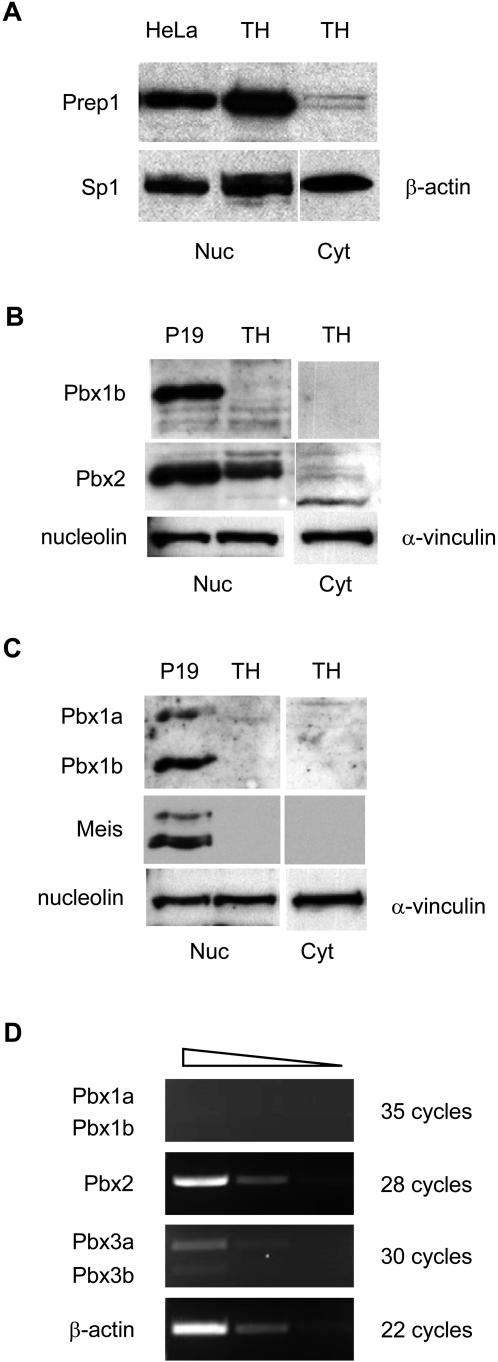
Analysis of expression of Prep1, Pbx, and Meis proteins in thymus of FVB/N wt mice. Nuclear (Nuc) and cytoplasmic (Cyt) extracts of thymocytes (TH) of FVB/N wild-type mice were blotted onto nitrocellulose membranes and incubated with anti-Prep1 (A), anti-Pbx1b and anti-Pbx2 (B), anti-Pbx1 (Pbx1a and Pbx1b) and anti-Meis (C) antibodies. Anti-Sp1, anti-nucleolin, anti-β-actin and anti-α-vinculin antibodies were used as loading controls for nuclear and cytoplasmic extracts. A nuclear extracts of HeLa cell line or P19 cell line induced with retinoic acid (10^−7^ M) for 24 h were used as positive controls for the expression of Prep1, Pbx1 and Meis proteins. (D) RT-PCR analysis of Pbx1 (Pbx1a and Pbx1b), Pbx2 and Pbx3 (Pbx3a and Pbx3b) expression. Total RNA was isolated from thymocytes of FVB/N mice. Serial dilutions (1∶5) of cDNA were amplified using primers specific for Pbx1, Pbx2 and Pbx3. Primers for β-actin were used as a control.

**Figure 2:**
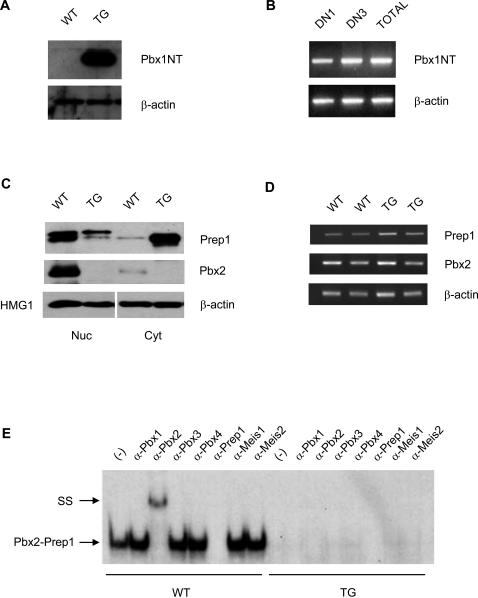
Analysis of expression of Pbx1NT transgene, Prep1 and Pbx2 proteins and Prep1-Pbx DNA-binding activity in Pbx1NT TG mice. (A) Western blot analysis of Pbx1NT expression in the thymus of *Pbx1NT TG mice*. Cytoplasmic extracts (30 µg) of total thymus of Pbx1NT TG mice (TG) and their wild-type controls (WT) were blotted onto PVDF membranes and incubated with anti-Pbx1 antibody. Anti-β-actin antibody was used as loading control. (B) RT-PCR analysis of Pbx1NT expression in sorted DN1, DN3 as well as in unsorted (TOTAL) thymocytes of *Pbx1NT TG mice*. Total RNA was isolated from the DN1, DN3 and total thymocytes and reverse transcribed. PCR amplification was performed using Pbx1NT-specific primers for 30 cycles and β-actin primers for 25 cycles. (C) Nuclear (Nuc) and cytoplasmic (Cyt) extracts of Pbx1NT TG mice (TG) and their wild-type controls (WT) were blotted onto PVDF membranes and incubated with anti-Prep1 and anti-Pbx2 antibodies. Anti-β-actin and anti-HMG1 antibodies were used as loading controls for cytoplasmic and nuclear fractions. (D) Total RNA was isolated from thymocytes of two Pbx1NT TG mice (TG) and their wild-type controls (WT) and reverse transcribed. PCR amplification was performed using Prep1 and Pbx2 -specific primers for 28 cycles and β-actin primers for 25 cycles. (E) Electrophoretic mobility shift assays were performed with the radiolabeled O1 double-stranded oligonucleotide, incubated with nuclear extracts from the thymi of wild-type (WT) or Pbx1NT TG (TG) mice without (−) or with antibodies against Prep1, Pbx1, Pbx2, Pbx3, Pbx4, Meis1 or Meis2. Pbx2-Prep1 arrow indicates the migration of complexes formed between Prep1 and Pbx2. SS, supershift.

As in the case of C57BL/6 thymocytes [3], Prep1, Pbx2, and Pbx3, but not Pbx1, Meis1, Meis2 and Meis3 are found in immature and mature wild type (wt) FVB/N T cells (Fig. 1). To investigate the relative contribution of cytosolic and nuclear Prep1 to T cell development we generated TG mice expressing the N-terminal fragment of a Pbx1 (lacking the homeodomain and nuclear localization signal) able to sequestrer Prep1 in the cytosol [5], under the control of the *lck* proximal promoter, which is active in T cells [9]. We produced two TG FVB/N lines expressing high (*Pbx1NTH*) and low (*Pbx1NTL*) levels of the *Pbx1NT* transgene, respectively. Immunoblot analysis indicated that *Pbx1NT* was expressed in transgenic thymi (Fig. 2A). Thymocyte subpopulations were sorted according to their developmental stage and transgene expression was analyzed by RT-PCR analysis. As shown in Fig. 2B, Pbx1NT mRNA was already expressed in the DN1 TG thymocyte population, continued to be expressed in DN3 and was clearly evident in the total thymocytes population. As similar results were obtained in the *Pbx1NTL* line with reduced protein level but comparable distribution of expression (not shown), all subsequent analyses were performed in the *Pbx1NTH* TG line (from here on defined as *Pbx1NT* TG mice).

### Pbx1NT causes cytoplasmic retention of Prep1 and a reduction of Pbx2 expression

Cytosolic and nuclear fractions of unfractionated thymocytes were analysed by immunoblotting with anti-Prep1 mAb. The relative representation of HMG1 in the nucleus and β-actin in the cytoplasm was used as loading control (Fig. 2C). While Prep1 was mostly found in the nucleus of wt thymocytes, it was mostly associated with the cytoplasm in cells expressing the *Pbx1NT* transgene (Fig. 2C).

We previously found that thymic *Pbx2* expression was decreased to almost undetectable levels in *Prep1^i/i^* hypomorphic mice [3]. Thus we analyzed whether the cytoplasmic retention of Prep1 by Pbx1NT would also result in abnormal *Pbx2* expression. Immunoblotting data showed a drastic reduction of nuclear Pbx2 in the TG when compared to wt thymocytes (Fig. 2C). Semi-quantitative RT-PCR assays showed that no difference in Pbx2 mRNA levels was observed between wt and TG thymus (Fig. 2D) indicating that reduced expression of *Pbx2* was likely due to a post-translational mechanism as observed elsewhere [10], [3], [4].

To analyze Prep1-Pbx-binding activity, EMSA experiments were performed (Fig. 2E). The composition of the DNA-binding complex was analyzed by supershift assays with antibodies against the different TALE family members. As in the case of C57BL/6 thymocytes [3], also in wt FVB/N thymocytes Prep1-Pbx2 dimers accounted for the vast majority of the Prep1-dependent DNA-binding activity, as only anti-Prep1 and anti-Pbx2 antibodies inhibited and supershifted the DNA-binding complex (Fig. 2E). Specific Prep1-dependent DNA-binding activity was instead absent in the nuclear extracts of *Pbx1NT* TG thymi (Fig. 2E), suggesting that no compensatory/residual DNA-binding activity exists in TG thymocytes.

Thus, the expression of *Pbx1NT* transgene results in cytoplasmic sequestration of Prep1, and causes the reduced expression of *Pbx2*, and the disappearance of Prep1-Pbx2 DNA-binding activity in developing thymocytes, as in the case of *Prep1^i/i^* hypomorphic mice [3].

### Immature DN3 and DN4 thymocytes accumulate in Pbx1NT TG mice, because of a higher rate of cells in S-phase

**Figure 3:**
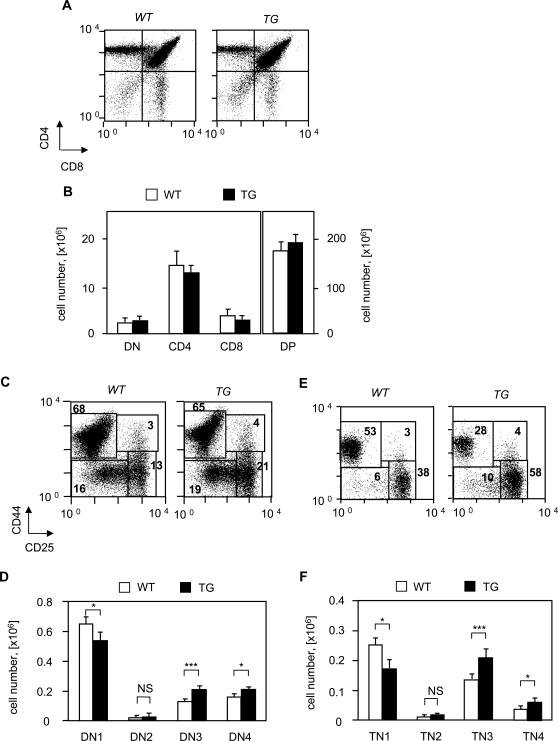
Flow cytometric analysis of thymocytes from Pbx1NT TG mice (TG) and their wild-type controls (WT). (A) Thymocytes were stained with PE-conjugated anti-CD4, CyChrome-conjugated anti-CD8, and FITC-conjugated anti-TCRβ antibodies. Viable cells were gated according to their forward scatter (FCS) and side scatter (SCS) characteristics. One representative of 8 independent experiments is shown. (B) Absolute numbers of DN, DP and SP (CD4^+^ and CD8^+^) cells. The cells were from wild-type mice (open bars) or from Pbx1NT TG mice (black bars). (C) Flow cytometric analysis of DN (CD4^−^CD8^−^) thymocytes from Pbx1NT TG mice and their wild-type controls. DN cells were enriched by rabbit anti-CD4 and anti-CD8 complement-mediated lysis (see Materials and Methods). DN thymocytes were stained with PE-conjugated anti-CD44, APC-conjugated anti-CD25 antibodies and CyChrome-conjugated anti-CD4 and anti-CD8 antibodies to negatively gate residual CD4 and CD8 -positive cells. Viable cells were gated according to their FCS and SCS characteristics. One representative of 8 independent experiments is shown. (D) Absolute numbers of DN1, DN2, DN3, and DN4 within DN cells. The cells were from wild-type mice (open bars) or from Pbx1ΝΤ TG mice (black bars). Values that were significantly different are indicated as follows: *, P<0.05; ***, P<0.001. (E) The same as in (C) for TN (CD4^−^CD8^−^CD3^−^) thymocytes. (F) The same as in (D) for TN thymocytes.

We next analyzed thymocyte representation in *Pbx1NT* TG mice by flow cytometry. The analysis of *Pbx1NT* TG and wt littermates demonstrated no difference in the thymus cellularity (205 +/−55×10^6^ versus 183×10^6^, respectively), and in the distribution of DN, DP and CD4 and CD8 SP thymocytes (Fig. 3A, B). Differences were instead observed within the DN (CD4^−^CD8^−^) or TN (CD3^−^CD4^−^CD8^−^) subsets (Fig. 3C–F). The frequency and total number of DN3 (CD44^−^ CD25^+^) and DN4 (CD44^−^ CD25^−^) thymocytes were increased by 50–100% in the TG mice when compared to wt littermates (Fig. 3C, D). Similar increase was also observed for TN3 and TN4 sub-populations (Fig. 3E, F). Interestingly, the increase in DN3 and DN4 cell number was first detectable at 6 weeks and became more pronounced with the mice aging (data not shown).

**Figure 4:**
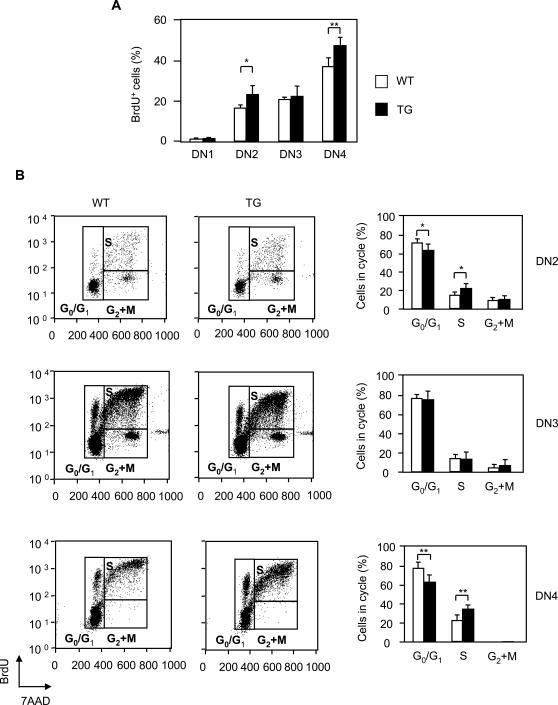
Analysis of proliferation of thymocytes of Pbx1NT TG mice. Percentage of BrdU-positive DN1, DN2, DN3 and DN4 thymocytes after *in-vivo* BrdU staining (see Matherial and Methods) is shown. The cells were from wild-type mice (open bars) or from Pbx1NT TG mice (black bars). (B) Cell cycle analysis of electronically gated DN2, DN3 and DN4 was performed with BrdU-flow kit (BD Biosciences, San Jose, CA) according to the manufacturer's recommendations. The cells were from wild-type mice (open bars) or from Pbx1ΝΤ TG mice (black bars).

The relative rate of proliferation and cell death, largely controlled by pre-TCR-driven signals, regulates the number of developing thymocytes [11]. Flow cytometry analysis of DN3 *Pbx1NT* TG and wt thymocytes revealed comparable surface TCR expression (not shown) suggesting that TCRβ chain rearrangement is not Prep1 or Pbx2 dependent. The rate of cell proliferation was determined by FACS analysis following *in vivo* BrdU i.p. administration. Cells in active cell division (in S phase) were found among all thymocyte subsets (Fig. 4A and not shown). When compared to wild type, DN2 and DN4 TG subpopulations revealed a lower frequency of cells in the G_1_ phase of the cell cycle, and a higher frequency of cells in the S phase (Fig. 4B). Thus, the increase of DN3 and DN4 cell numbers can be accounted for by a higher proliferation rate of DN2 and DN4 precursors.

### Pbx1NT TG mice have normal numbers of mature T cells

**Figure 5:**
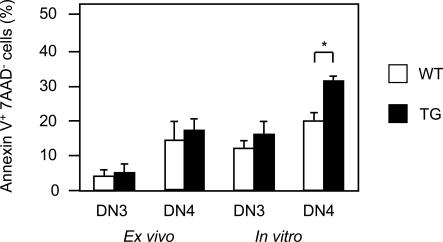
Analysis of apoptosis of thymocytes of Pbx1NT TG mice. Purified DN thymocytes from Pbx1ΝΤ TG mice and their wild-type controls were stained with PE-conjugated anti-CD44, APC-conjugated anti-CD25, FITC-conjugated annexin V, and 7AAD prior to FACS analysis. The percentage of annexinV-positive 7AAD-negative DN3 and DN4 thymocytes *ex vivo* (0h) and after 24 hours of incubation at 37°C (24 h) is shown. The cells were from wild-type mice (open bars) or from Pbx1ΝΤ TG mice (black bars). Values that were significantly different are indicated as follows: *, P<0.05.

In TG mice, developing thymocytes do not accumulate behind the DN4 stage of differentiation. We thus investigated whether a relative higher rate of cell death might account for the normalization of the cell number above the DN stage. Cell death was analyzed *ex vivo*, at the time of sacrifice and after 24 h of culture (*in vitro*) by annexin V and 7AAD staining and flow cytometry analysis. While a comparable frequency of apoptotic DN3 cells were observed in *Pbx1NT* TG and control wt littermates, a higher frequency was instead observed within the cultured *Pbx1NT* TG DN4 thymocytes (Fig. 5). Thus, the increased apoptotic rate of DN4 TG thymocytes might account for the failure of TG thymocytes to accumulate behind the DN4 stage.

In spite of the expression of *Pbx1NT* transgene in more mature thymocytes, and peripheral cells (not shown), the frequency and total number of DP and SP thymocyte and T cell were comparable in TG and wt littermates (Table 1).

**Table 1:** T cell populations in wild-type (wt) and *Pbx1NT* (tg) mice

Organ	Genotype	Cell number*	CD4^+^ cell number*	CD4^+^ percentage	CD8^+^ cell number*	CD8^+^ percentage	γδ TCR^+^ number*
Thymus n = 10	wt	**183.2** (45)	**13.9** (2.9)	**7.6** (1.1)	**3.5** (1.2)	**1.9** (0.7)	**0.40** (0.1)
	tg	**205.4** (55)	**12.1** (1.3)	**6.1** (0.9)	**2.6** (0.9)	**1.3** (0.2)	**0.35** (0.11)
Spleen n = 8	wt	**156.4** (21.7)	**9.6** (5.3)	**27.3** (3.0)	**13.2** (2.7)	**11.9** (1.5)	**N.D.**
	tg	**129.0** (43.5)	**27.1** (6.1)	**23.8** (2.9)	**10.2** (3.1)	**11.3** (1.0)	
Lymph nodes n = 8	wt	**22.9** (5.1)	**12.1** (3.4)	**53.7** (2.0)	**4.2** (2.3)	**18.5** (1.9)	**N.D.**
	tg	**24.8** (5.5)	**12.5** (2.5)	**50.7** (3.5)	**5.2** (0.9)	**21.0** (2.7)	

Data are means (bold)±s.e.m. (in parenthesis)

* million of cells per organ

N.D. non determined

## Discussion

Here we analyzed the effect of the cytoplasmic retention of Prep1 by the lck-driven expression of a TG N-terminal fragment of Pbx1, and found that mislocalization of Prep1 has an impact on *Pbx2* expression and on early stages of thymocyte development, causing the transient increase of DN3/DN4 cell numbers.

We previously found that the reduced Prep1 level in hypomorphic *Prep1^i/i^* mice caused an abnormal thymic development revealed by an increase of CD4^−^ CD8^−^ DN thymocytes, and a decrease in CD4^+^ and CD8^+^ SP cells, mirrored by a lower number of circulating T cells. In this study, DN, DP and SP precursors appeared to be normally represented in *Pbx1NT* TG mice. However, further analysis of DN subpopulations revealed an increase of DN3 and DN4 subpopulations best explained by the higher *in vivo* proliferation rate of DN2 and DN4 TG subpopulations. Thus the accumulation of DN cell subsets closely resembles the one found in the *Prep1^i/i^* mice, in which the higher rate of DN cell proliferation was not revealed, possibly because of the further differentiation defects. In spite of the increased number of proliferating DN2 and DN4 precursors, the total number of DN cells was comparable in *Pbx1NT* TG and wt littermates, and normal T cell development appeared to be preserved at later stages. This is best explained by the observation that *Pbx1NT* TG DN thymocytes had a higher propensity to undergo cell death when compared to control cells. Thus a higher rate of cell proliferation, and of cell death might account for the transient developmental defects observed in *Pbx1NT* TG mice. The Prep1 dependent molecular events underlying these events remain to be fully elucidated.

The thymic and peripheral immune phenotypes of *Pbx1NT* TG and *Prep1^i/i^* mice were not completely overlapping, although in either case Prep1-dependent DNA binding activity was absent. In particular, while earlier stages of thymic development were impaired in both *Prep1^i/i^* hypomorphic and *Pbx1NT* TG mice, thymocyte maturation beyond the DN stage was normal in *Pbx1NT* mice but not in *Prep1^i/i^* hypomorphic mice. Furthermore, while the number of mature T cells was significantly reduced in *Prep1^i/i^* hypomorphic mice [3], it was normal in *Pbx1NT* mice. The reasons for the diversities could be explained by the fact that the *Pbx1NT* transgene is controlled by the proximal Lck promoter, which might be less active in mature T cells. However, we found that *Pbx1NT* expression was detectable in the periphery. It is important to point out that in *Prep1^i/i^* hypomorphic mice *Prep1* expression was reduced to a very low level in all cellular compartments, while in *Pbx1NT* TG mice Prep1 representation was reduced in the nucleus, but not in the cytoplasm. Thus while genetic inactivation of the *Prep1* gene might abrogate Prep1 (and Pbx2) transcriptional activity, the functional sequestration of the protein might favor some unknown cytoplasmic functions. We thus favor the possibility that Prep1 plays multiple roles during maturation and/or that nuclear and cytoplasmic Prep1 have different functions.

As in the case of *Prep1* hypomorphic mice, functional inactivation of Prep1 (by cytosolic sequestration) caused a reduction in the levels of Pbx2 protein. This was likely due to a reduction of protein stability, as comparable Pbx2 mRNA levels were found, and possibly due to increased proteasomal degradation [10]. The finding that Pbx2 was reduced to comparable extents in *Pbx1NT* and *Prep1^i/i^* hypomorphic mice, which only share some of the abnormalities of thymic development, suggests that Prep1 and Pbx2 might have non redundant roles and/or Prep1 might exert a Pbx2-independent function in determining the T cell fate.

How Prep1, Pbx2 and/or Prep1/Pbx2 dimers control early stages of T cell development remains to be elucidated. We found that surface expression of the pre-TCR and the intracellular expression of the TCRβ are normal in *Pbx1NT* TG thymocytes. Thus, these transcription factors do not seem to participate to early T cell commitment. However, they might be targeted by pre-TCR-driven events. Accordingly, both proliferation and survival of developing thymocytes appeared to be altered in the absence of functional Prep1 and/or Pbx2. It is also possible that Prep1 and/or Pbx2 control IL-7 responsiveness, the most important pro-survival cytokine at DN2 stage [12], [13]. Although we did not notice any significant changes of IL-7R expression in *Prep1^i/i^* hypomorphic mice [3], we cannot exclude that such changes can occur in *Pbx1NT* TG mice. Alternatively, Prep1 may play a role at some steps immediately downstream of IL-7 activation.

In conclusion, our data complement the analysis of *Prep1* hypomorphic mice, and further support a role for Prep1 and/or Pbx2 in determining T cell fate.
